# *Notes from the Field*: Lead and Cadmium Exposure in Electronic Recyclers — Two States, 2015 and 2017

**DOI:** 10.15585/mmwr.mm6807a4

**Published:** 2019-02-22

**Authors:** Reed Grimes, Catherine Beaucham, Jessica Ramsey

**Affiliations:** ^1^Epidemic Intelligence Service, CDC; ^2^Division of Surveillance, Hazard Evaluations, and Field Studies, National Institute for Occupational Safety and Health, CDC.

In 2012, CDC’s National Institute for Occupational Safety and Health (NIOSH) became aware of the potential for occupational and take-home exposures to lead and cadmium in the electronics recycling industry (*1*) and contacted electronics industry stakeholders to discuss these exposures and provide information about NIOSH’s ability to investigate workplace hazards. NIOSH subsequently received requests for health hazard evaluations to estimate employee exposures and to assess the potential for take-home contamination from lead and cadmium at facilities A and B, in two states. Both facilities refurbished electronics for resale, removed electronic components for reuse and resale, and recycled electronics for waste management. Facility A employees received and inventoried electronics for either recycling or refurbishment and resale. Recycled materials were disassembled by hand or shredded using an industrial shredder. Employees at facility B performed similar job tasks as did those at facility A, but did not shred electronic parts.

NIOSH evaluated facility A over 3 days in August 2015 and facility B over 2 days in July 2017. The evaluations included collection of 1) blood specimens to assess worker uptake of lead and cadmium; 2) personal air samples for assessment of lead and cadmium exposure; 3) questionnaire data concerning demographics, hand hygiene, and use of designated work clothing and personal protective equipment; 4) end-of-shift hand wipes to assess dermal exposure; and 5) surface wipe samples on nonprocessing surfaces, such as refrigerator handles and microwave control panels. All 15 employees at facility A and eight of 12 employees at facility B took part in the assessment (Table).

**TABLE:** Lead and cadmium testing results at two electronic recycling facilities — two states, 2015 and 2017

Characteristic	No. (%)	
	Facility A* (15 employees)	Facility B (12 employees)
**Total participants**	**15 (100)**	**8 (75)**
Male	12 (75)	7 (88)
Current smoker	6 (40)	5 (63)
Age (yrs), median (range)	37 (20–52)	32 (19–47)
Months working at facility, median (range)	15 (1–88)	27 (2–66)
**Personal air results**^†^ **exceeding OELs^§^**		
Cadmium	1/45 (2)	0/16 (0)
Lead	0/45 (0)	0/16 (0)
**Blood levels above reference ranges^¶^**		
Cadmium	0/12 (0)	0/8 (0)
Lead	3/12 (25)	0/8 (0)
**Positive end-of-shift dermal wipes**		
Cadmium	14/14 (100)	8/8 (100)
Lead	14/14 (100)	8/8 (100)
**Survey results of work practices**		
Hand washing practiced all or most of time	12/15 (80)	6/8 (75)
Wear gloves all or most of the time	7/15 (47)	6/8 (75)
Wear work clothes home	12/15 (80)	7/8 (88)
Wear work shoes home	13/15 (87)	6/8 (75)

**Abbreviation:** OEL = occupational exposure limit.

* Some participants at facility A elected not to participate in every part of the evaluation.

^†^ One sample per day over 3 days for facility A, and one sample per day over 2 days for facility B.

^§^ OELs over an 8-hour time-weighted average: lead = 50 *μ*g/m^3^; cadmium = 5 *μ*g/m^3^.

^¶^ Reference ranges: blood lead = 5 *μ*g/dL; blood cadmium = 5 *μ*g/L.

Only facility A employees were found to have blood lead levels above the CDC reference level of 5 *μ*g/dL. Lead in personal air samples ranged from undetectable to 19 *μ*g/m^3^ (facility A), and from undetectable to 0.59 *μ*g/m^3^ (facility B); no samples at either facility exceeded the lead occupational exposure limit (OEL) of 50 *μ*g/m^3^.

Employee blood cadmium levels at both facilities were below the American Conference of Governmental Industrial Hygienists Biologic Exposure Index of 5 *μ*g/L. One facility A employee’s personal air cadmium exceeded the NIOSH-recommended OEL of 5 *μ*g/m^3^. Cadmium in personal air samples ranged from undetectable to 6.4 *μ*g/m^3^ (facility A) and from undetectable to 0.05 *μ*g/m^3^ (facility B). All participating employees at both facilities had both cadmium and lead on their hands after washing them at the end of their shift. Nonprocessing surfaces at facility A were contaminated with cadmium (nine of 12) and lead (11 of 12), as were surfaces at facility B (12 of 13 and 13 of 13, respectively).

Employees at both facilities reported wearing cloth, nitrile, and cut-resistant gloves. More facility B employees reported wearing gloves all or most of the time while at work than did facility A employees. All employees who wore gloves reused them. Employees were permitted to wear their work clothes and shoes home, and neither facility had capability for onsite laundering of work clothing. Employees at both facilities performed dry sweeping of surfaces, which can reaersolize metal-containing dust.

The findings of these health hazard evaluations confirm workplace exposures to lead and cadmium at these facilities and suggest that employees in shredding facilities might be at higher risk for exposure than are those at nonshredding facilities. The presence of lead and cadmium on the hands of employees at both facilities after end-of-shift handwashing highlights the potential for take-home contamination.

Adverse health effects have been reported in persons chronically exposed to lead even at levels at or below the CDC reference level of 5 *μ*g/dL (*2*,*3*), as well as in those with long-term cadmium exposure (*4*). Electronic recycling employers should maintain written prevention programs that emphasize evaluating and reducing employees’ exposure to hazardous metals. To further reduce exposure to hazardous metals, employers could implement strict handwashing policies with lead-removing soap to prevent take-home exposure, perform routine housekeeping to prevent build-up of hazardous heavy metals, and prohibit dry sweeping to prevent exposure to reaerosolized metals. Employers should ensure that employees wear gloves during disassembly and shredding of electronics, and if cut-resistant or cloth gloves are re-used, clean inner gloves should be provided. More detailed information in health hazard evaluation reports for facility A and facility B is available (*5*,*6*).
